# Chitosan Cryogels Cross-Linked with 1,1,3-Triglycidyloxypropane: Mechanical Properties and Cytotoxicity for Cancer Cell 3D Cultures

**DOI:** 10.3390/biomimetics8020228

**Published:** 2023-05-29

**Authors:** Yuliya Privar, Andrey Boroda, Alexandr Pestov, Daniil Kazantsev, Daniil Malyshev, Anna Skatova, Svetlana Bratskaya

**Affiliations:** 1 Institute of Chemistry, Far Eastern Branch of the Russian Academy of Sciences, 159, Prospekt 100-Letiya Vladivostoka, 690022 Vladivostok, Russia; 2 A.V. Zhirmunsky National Scientific Center of Marine Biology, Far Eastern Branch of Russian Academy of Sciences, 17, Palchevskogo Street, 690041 Vladivostok, Russia; borodandy@gmail.com (A.B.);; 3 Postovsky Institute of Organic Synthesis, Urals Branch of the Russian Academy of Sciences, 22, S. Kovalevskoy Street, 620990 Ekaterinburg, Russia; pestov@ios.uran.ru (A.P.);

**Keywords:** chitosan, cryogel, cross-linking, mechanical properties, triglycidyl ether, cytotoxicity, stiffness

## Abstract

Here, we have presented a new method of 1,1,3-triglycidyloxypropane (TGP) synthesis and investigated how cross-linker branching affects mechanical properties and cytotoxicity of chitosan scaffolds in comparison with those cross-linked using diglycidyl ethers of 1,4-butandiol (BDDGE) and poly(ethylene glycol) (PEGDGE). We have demonstrated that TGP is an efficient cross-linker for chitosan at a subzero temperature at TGP:chitosan molar ratios from 1:1 to 1:20. Although the elasticity of chitosan scaffolds increased in the following order of the cross-linkers PEGDGE > TGP > BDDGE, TGP provided cryogels with the highest compressive strength. Chitosan-TGP cryogels have shown low cytotoxicity for colorectal cancer HCT 116 cell line and supported the formation of 3D multicellular structures of the spherical shape and size up to 200 µm, while in more brittle chitosan-BDDGE cryogel this cell culture formed epithelia-like sheets. Hence, the selection of the cross-linker type and concentration for chitosan scaffold fabrication can be used to mimic the solid tumor microenvironment of certain human tissue, control matrix-driven changes in the morphology of cancer cell aggregates, and facilitate long-term experiments with 3D tumor cell cultures.

## 1. Introduction

A growing field of research based on microphysiological systems involving organoids/spheroids or patient-derived tumor cells has become a step toward personalized medicine [1]. Different types of polymeric scaffolds have been used to develop 3D tumor models for in vitro tests of anticancer drugs, investigation of cancer progression, and cell–cell interactions in co-culture [2,3,4,5]. Such scaffolds should facilitate cancer cells to form 3D multicellular spheroids, which mimic the solid tumor in vivo [6], and offer an environment in which the alterations of tumor cell phenotype are similar to those associated with tumor progression in vivo [7]. To meet this challenge, both the chemical composition and stiffness of the scaffolds must be finely tuned to the specific cancer cell culture.

Interest in chitosan as a matrix for 3D tumor models is determined by the structural similarity of chitosan to glycosaminoglycans, which are important components of the extracellular matrix (ECM) of different tissues [8]. Furthermore, experimental results have shown that culturing cancer cells in chitosan-based scaffolds increased cell motility, drug resistance, quiescent population, self-renewal capacity, and stemness [6].

The high stiffness of ECM is known to promote more aggressive and metastatic phenotypes in several cancers [9,10,11]. However, hydrogels resembling the stiffness of such tumors’ microenvironment have low permeability for nutrients that negatively affects the size, morphology, and density of the growing 3D cell aggregates [12,13]. The optimal alternative to the stiff polymeric hydrogels is macroporous scaffolds, for which stiffens of the pore walls up to hundreds of kPa can be reached without sacrificing the efficiency of transport of nutrients and cell metabolites [14].

Fabrication of chitosan scaffolds with a broad range of stiffness is complicated by the solubility of this polymer only in acidic media, where a selection of efficient cross-linkers is limited to aldehydes, which are toxic when used at high concentrations [15]. The problem of toxicity of stiff chitosan scaffolds was eliminated via lyophilization of polyelectrolyte complexes of chitosan with oppositely charged polysaccharides [16,17,18]. However, this approach affects not only the stiffness but also the chemistry of the scaffolds.

Recently, we have suggested another solution to fabricate compressible shape-recovering macroporous chitosan scaffolds using diglycidyl ethers of glycols as a cross-linker in acidic media at a subzero temperature [19]. Using this fabrication method morphology and mechanical properties of chitosan scaffolds for 3D cancer models can be varied in a broad range using diglycidyl ethers with different chain lengths [20,21]. To extend this approach and fabricate flexible chitosan scaffolds with higher compressive strength, we have suggested here to use 1,1,3-triglycidyloxypropane (TGP) as a cross-linking agent for chitosan at subzero temperature.

**Scheme 1:**
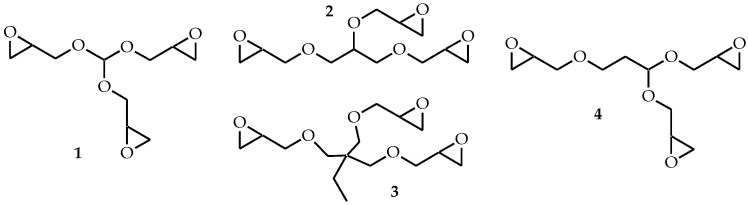
Chemical structure of 1,1,3-tri(glycidyloxy)methane (1), 1,2,3-tri(glycidyloxy)propane or glycerol triglycidyl ether (2), 1,1,1-triglycidyltrimethylolpropane (3), and 1,1,3-triglycidyloxypropane (4).

In general, different types of triglycidyl ethers (Scheme 1, structures 1–3) have been used as cross-linkers to improve the mechanical properties of chitosan-based materials. Higher probability of cross-linkage formation at lower concentrations of branched cross-linkers, which is also important to reduce cytotoxicity, inspired screening triglycidyl ethers efficiency in comparison with diglycidyl ethers. Tri(glycidyloxy)methane (Scheme 1, structure 1) was used as a cross-linking solvent of polyphenolic resins [22]. Glycerol triglycidyl ether (Scheme 1, structure 2) is the most frequently reported reagent of this type. It was used alone [23] or in combination with other glycidyl ethers [24]. Products of its polymerization were suggested for cell immobilization [25] and tissue engineering [26]. Glycerol triglycidyl ether was used as a cross-linker to fabricate inflammation stimulus-responsive degradable for hyaluronic acid matrixes for implantable cellular DNA-delivery systems [27] and fixation of biological tissues [28]. Chromium-free tanning agent with antimicrobial properties was synthesized using oligomeric chitosan and glycerol triglycidyl ether as an eco-friendly reagent for leather treatment [29]. Crosslinking of gelatin with glycerol epoxy resin to form a hyperbranched polymer was shown to reduce its brittleness and increase water resistance [30]. 1,1,1-triglycidyltrimethylolpropane (Scheme 1, structure 3) was applied to improve the mechanical properties of chitosan beads precipitated in alkaline solution [31], fabricate chitosan nanofiltration membranes [32], and graft chitosan to the plasma-treated polypropylene fabric [33]. In all cases, reactions proceeded at room or elevated temperature, and the advantage of triglycidyl over diglycidyl ethers was not always unquestionable due to the higher probability of self-cross-linking reaction at high concentration of triglycidyl ethers [33].

Methods of triglycidyl ether synthesis were earlier reported mainly in patent literature. Here, we have suggested a new method of 1,1,3-triglycidyloxypropane (Scheme 1, structure 4) synthesis and investigated how cross-linker branching affects mechanical properties and cytotoxicity of chitosan cryogels in comparison with scaffolds cross-linked using diglycidyl ethers of 1,4-butandiol and poly(ethylene glycol).

## 2. Materials and Methods

### 2.1. Materials

Low molecular weight (30 kDa) chitosan with deacetylation degree of 90% was purchased from BioLog Heppe GmbH (Landsberg, Germany). Other reagents were purchased from Sigma-Aldrich (St. Louis, MO, USA) and used as received.

### 2.2. Synthesis of 1,1,3-Triglycidyloxypropane (TGP)

1,1,3-Triallyloxypropane. A mixture of 5.25 g (0.0936 mol) of acrolein, 19.16 mL (0.2817 mol) of allyl alcohol, 40 mL of chloroform, and 0.34 g (1.97 mmol) of p-toluenesulfonic acid was reflux with a Dean-Stark trap until the formation of 1.7 mL of water. Then, the reaction mass was washed with 10% sodium carbonate solution and the organic layer was dried over anhydrous sodium sulfate and fractionated in a vacuum, collecting the product at 90–100 °C (1 Torr). Yield 73%. Found, %: C 67.81; H 9.47. Calculated, %: C 67.89; H 9.50. NMR ^1^H, CDCl_3_, ppm: 1.95 (q, 2H, *J* = 6.27, 12.39, CH_2_), 3.52 (t, 2H, *J* = 6.68, CH_2_), 4.04 (m, 6H, CH_2_), 4.78 (t, 1H, *J* = 5.93, CH), 5.13–5.30 (m, 6H, =CH_2_), 5.91 (m, 3H, =CH-). FT-IR, cm^−1^: 3080, 2954, 2931, 2875, 1460, 1492, 934, 1004, 1053.

Bromohydrin of 1,1,3-triallyloxypropane. A mixture of 3 g (0.014 mol) of 1,1,3-triallyloxypropane, 18.39 mL of water, and 7.70 g of N-bromosuccinimide (0.043 mol) was stirred for 4 h at 60 °C. Then, the reaction mass was extracted with methylene chloride in 10 mL × 3, the organic layer was dried over anhydrous sodium sulfate, and methylene chloride was distilled off on a rotary evaporator. Yield 95%. Found, %: C 28.34; H 4.53; Br 48.25. Calculated, %: C 28.65; H 4.61; Br 47.65. NMR ^1^H, CDCl_3_, ppm: 1.96 (q, 2H, *J* = 6.28, 12.40, CH_2_), 3.45–3.67 (m, 14H, CH_2_), 4.22 (m, 3H, CH-OH), 5.04 (t, 1H, *J* = 6.01, CH), 5.28 (s, 3H, OH). FT-IR, cm^−1^: 2934, 2883, 1424, 1222, 1156, 1151, 1031, 1064.

1,1,3-Triglycidyloxypropane (TGP). A mixture of 4.0 g (7.95 mmol) of bromohydrin of 1,1,3-triallyloxypropane, 0.88 g (0.022 mol) of sodium hydroxide, and 5 mL of water was stirred at room temperature (RT) for 35 min. Then the reaction mass was extracted with methylene chloride in 10 mL × 4, the organic layer was dried over anhydrous sodium sulfate, and methylene chloride was distilled off on a rotary evaporator. Yield 83%. Found, %: C 55.21; H 7.64. Calculated, %: C 55.37; H 7.75. NMR ^1^H, CDCl_3_, ppm: 1.90 (q, 2H, *J* = 6.29, 12.26, CH_2_), 2.58 (m, 3H, CH_2_), 2.77 (m, 3H, CH_2_), 3.12 (m, 3H, CH), 3.12 (m, 3H, CH), 3.41–3.85 (m, 8H, CH_2_), 4.77 (t, 1H, *J* = 5.76, CH) FT-IR, cm^−1^: 2998, 2962, 2928, 2877, 1256, 1223, 1099, 1044.

### 2.3. Fabrication of Cryogels

The 3%-chitosan solution was prepared in hydrochloric acid at equimolar NH_2_:HCl ratio and pH was adjusted to 5. The calculated amounts of the 10% solution of TGP in dimethyl sulfoxide, corresponding to the molar ratios of TGP:chitosan 1:4 and 1:12 were added to the chitosan solutions and sonicated for 30 s using Bandelin Sonopuls UW 3200 Ultrasonic Homogenizer with KE76 tip (Bandelin, Berlin, Germany). Then, solutions were immediately placed into the 2 mL plastic syringes and kept in a freezer (Liebherr, Kirchdorf an der Iller, Germany) at −10 °C for 7 days. After thawing, the cryogels were washed with distilled water using a peristaltic pump (Ismatec, Wertheim, Germany) at the flow rate of 5 bed volumes/h to remove unreacted chemicals.

### 2.4. Characterization

#### 2.4.1. Structure Analysis

^1^H NMR spectra were recorded on a Bruker AVANCE-500 spectrometer. Fourier transform infrared (FT-IR) spectra were recorded using an IR Affinity-1 spectrometer with QATR 10 single reflection ATR accessory (Shimadzu, Kyoto, Japan).

#### 2.4.2. Chitosan Gelation, Mechanical Properties and Swelling of Cryogels

Gelation time in 3% chitosan solution at equimolar TGP:chitosan ratio and mechanical properties of the hydrogels and cryogels were investigated using a Physica MCR 301 rheometer (Anton Paar GmbH, Graz, Austria) with a plate–plate measuring system with a diameter of 25 mm. Frequency sweep curves were recorded in the range between 0.1 and 100 Hz at a temperature of 25 °C at constant strain of 5% (which was within the linear viscoelastic region). The dependence of the storage (G′) and loss (G″) moduli at a frequency of 11.2 Hz vs. time was used to determine the gelation time. Strain–stress curves were recorded for the cylindrically shaped cryogels with a diameter of 9 mm and height of 9 mm at a constant speed of 0.01 mm/s.

Swelling of the cryogels was determined from the difference in weights of the swollen and dry material (the measurements were performed for freshly prepared cryogels from wet to dry state).

#### 2.4.3. Cell Cultivation and Cytotoxicity Analysis

The 24-well culture plates (TPP, Trasadingen, Switzerland) and cryogel disks were used to grow HCT 116 cells (Sigma-Aldrich, St. Louis, MO, USA) in adhesive and 3D conditions (in cryogel disks 7–9 mm in diameter and 4 mm thick), consequently.

HCT 116 cells were seeded in adhesive plates at a density of 10 × 10^3^ cells/well in 1 mL of Dulbecco’s modified Eagle’s medium (DMEM, #12800017, Gibco™, Thermo Fisher Scientific, Altrincham, UK) supplemented with 10% (*v*/*v*) fetal bovine serum (HyClone, Logan, UT, USA), 3.7 mg/mL sodium bicarbonate (Sigma-Aldrich), 1× mixture of non-essential amino acids (Waltham, MA, USA, Gibco), 100 U/mL penicillin (Gibco), and 100 µg/mL streptomycin (Gibco).

The fabricated cryogel was cut into disks, and each disk was placed in well of a 24-well TPP culture plate, which was consistently washed with 5 mL of Dulbecco’s phosphate buffer saline (DPBS, Sigma-Aldrich) without Ca^2+^ and Mg^2+^, and 5 mL of DMEM with additives. Free liquid was aspirated from the cryogel disks and the HCT 116 cell suspension was evenly applied on the top round surface of the disks at a density 10 × 10^3^ cells/disk in 100 µL of DMEM with mechanical pipette Research^®^ plus (Eppendorf, Hamburg, Germany) equipped with a 200 µL tip and cultivated at +37 °C, 5% CO_2,_ and 90% relative humidity. The medium has been replaced every 2 days.

The cells were monitored daily under a CKX41 inverted microscope (Olympus, Shinjuku City, Tokyo, Japan) equipped with phase-contrast optics and imaged with an Axiocam 105 color digital camera (Carl Zeiss, Oberkochen, Germany) in ZEN 2 (blue edition, Carl Zeiss).

The viability and functional activity of cells were analyzed after 3, 7, 10, 14, and 33 days of cultivation: the cryogel disks were washed with 1 mL of DPBS, HCT 116 cells and their spheroids were disaggregated and detached by incubation with 1 mL of 0.05% (*w*/*v*) trypsin—0.02% (*w*/*v*) EDTA solution (Sigma-Aldrich) from the wells or cryogel disks for 2 h at +37 °C, and centrifuged at 500× *g* for 5 min. A pellet of trypsinized cells from a single well of a 24-well plate or cryogel disk was re-suspended in 100 µL of DPBS with 10 µM 2′,7′-dichlorodihydrofluorescein diacetate (H_2_DCFDA, Sigma-Aldrich) to assess the mitochondrial activity, and 1 µg/mL 4′,6′-diamidino-2-phenylindole (DAPI, Sigma-Aldrich) to stain dead cells. The cell suspension was incubated at room temperature in the dark for 10 min and then diluted with 150 µL of DPBS before analysis with a CytoFLEX S flow cytometer (Beckman-Coulter, Brea, CA, USA) connected to a computer running CytExpert software (version 2.5, Beckman-Coulter). A detailed description of cell cultivation and flow cytometrical analysis is given in [34]. The algorithm of analysis of flow cytometrical data of stained cells is shown in Figure S1 (Supplementary Materials).

## 3. Results and Discussion

### 3.1. Synthesis of 1,1,3-Triglycidyloxypropane (TGP)

TGP was obtained via the successive treatment of acrolein with allyl alcohol yielding 1,1,3-triallyloxypropane, which was further converted to triglycidyl ether.

**Scheme 2:**
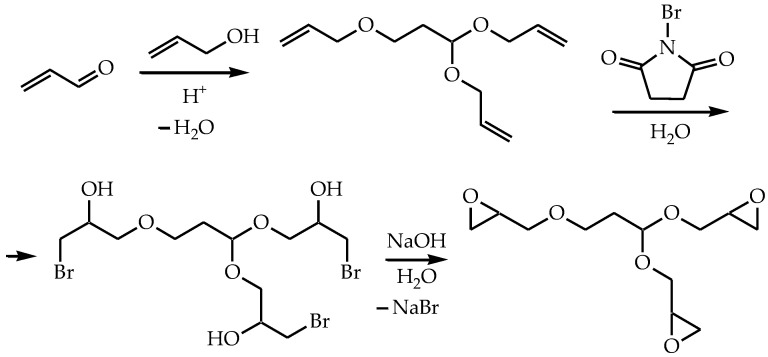
Synthesis of TGP.

Acrolein interaction with allyl alcohol proceeds via nucleophilic Michael addition reaction with the formation of an allyloxypropyl group and via nucleophilic addition-elimination reaction with the formation of an acetal functional group. Further conversion of the obtained 1,1,3-triallyloxypropane to the corresponding bromohydrin was carried out using N-bromosuccinimide. The oxirane rings were formed via the bromo derivative treatment with sodium hydroxide (Scheme 2).

Conversion of 1,1,3-triallyloxypropane to bromohydrin and triglycidyl ether is complicated by the high hydrophilicity of the products and intermediates, but the developed synthesis method can be scaled up for the preparative production of TGP with a total yield of 57%.

### 3.2. Mechanism of Chitosan Cross-Linking with TGP

Taking into account our previous experience in chitosan cross-linking with ethylene glycol diglycidyl ether (EGDGE) [19], 1,4-butanediol diglycidyl ether (BDDGE) [20] and poly(ethylene glycol) diglycidyl ether (PEGDGE) [19,21], gelation of chitosan in solution after TGP addition was first investigated at RT, pH 5, and TGP:chitosan mole ratio of 1:1. Then, cryogels were fabricated in a broader range of TGP:chitosan mole ratios at −10 °C.

Important feature of TGP in comparison with diglycidyl ethers and glycerol triglycidyl ether, earlier used for chitosan cross-linking, is the presence of an acetal functional group, which can be hydrolized to aldehyde group. Thus, TGP can be considered as a heterofunctional cross-linker, which can interact with chitosan via a dual cross-linking mechanism.

**Figure 1:**
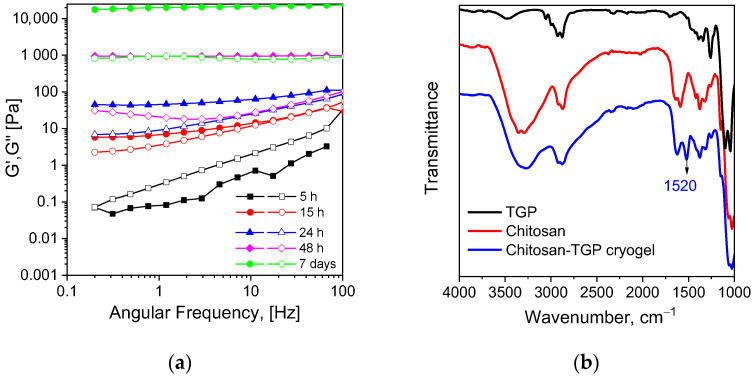
Time-dependent mechanical spectra of 3% chitosan solution after addition of equimolar content of TGP at T = 25 °C: filled symbols —storage moduli (G′), open symbols—loss moduli (G″)—(**a**). FT-IR spectra of TGP, chitosan, and chitosan cryogel cross-linked with TGP at molar ratio TGP:chitosan of 1:4 (**b**).

The evolution of loss (G″) and storage (G′) moduli (Figure 1a) indicated gel formation in chitosan-TGP solution between 10 and 15 h. After seven days of gelation at RT storage modulus of chitosan-TGP hydrogel reached 25 kPa that was notably higher in comparison with chitosan hydrogels cross-linked with EGDGE (4 kPa) and PEGDGE (12 kPa) under the same conditions [19]. It shows that the reaction of triglycidyl self-cross-linking, which was reported to decrease the efficiency of chitosan grafting to polypropylene fabrics using 1,1,1-triglycidyltrimethylolpropane in comparison with diglycidyl ether of ethylene glycol [33], was not predominant under the selected cross-linking conditions.

Despite the higher cross-linking efficiency of TGP in comparison with EGDGE, BDDGE, and PEGDGE at RT, we did not observe a significant reduction of the scaffold fabrication time at subzero temperature (after three days cryogels cross-linked at equimolar TGP:chitosan ratio had swelling over 20,000% and low mechanical stability). However, mechanically stable shape-recovering cryogels were obtained in preliminary experiments at all TGP:chitosan mole ratios from 1:1 to 1:20 at a reaction time of seven days. It should be mentioned that, although 1,1,1-triglycidyltrimethylolpropane showed higher efficiency than diglycidyl ether of ethylene glycol for chitosan beads cross-linking at mole ratio to chitosan of 1:10, the higher cross-linker concentration (mole ratio of 1:2) was required to avoid cracks of hydrogel in sorption application [31]. This demonstrates the significant advantage chitosan cross-linking with triglycidyl ether under subzero conditions to fabricate flexible crack-free materials for versatile applications. Detailed analysis of the swelling and mechanical properties of chitosan-TGP cryogels in comparison with earlier reported cryogels cross-linked with diglycidyl ethers [21] is presented in Section 3.3.

**Scheme 3:**
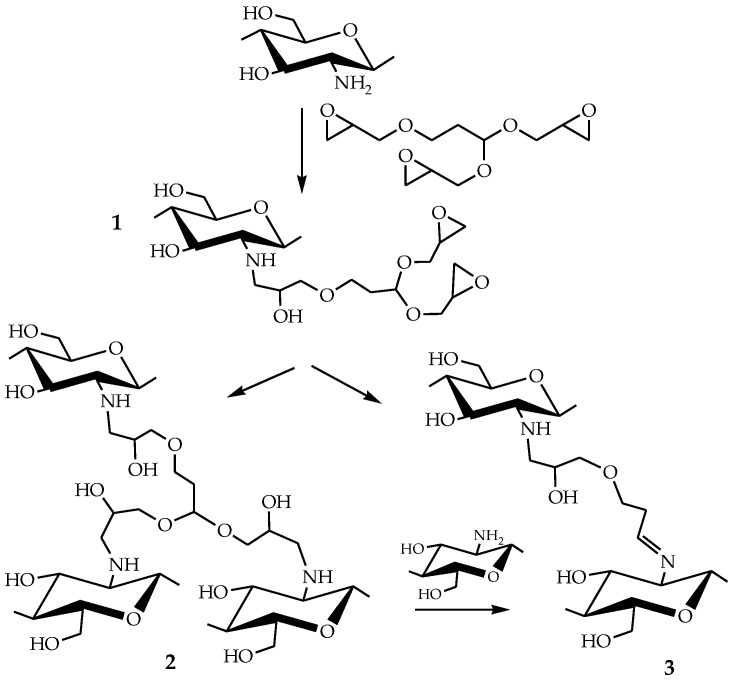
Mechanism of chitosan cross-linking with TGP.

The mechanism of chitosan cross-linking with TGP was investigated using FT-IR spectroscopy. In comparison with chitosan cryogels cross-linked in an acidic medium using diglycidyl ethers of glycols [19], where interactions via epoxy-ring opening reactions with amino and hydroxyl groups of chitosan were confirmed (Scheme 3, structure 2), a new untypical band at 1520 cm^−1^ appeared in FT-IR spectrum of chitosan-TGP cryogels (Figure 1b). This band can be assigned to the protonated imine bond (C=N^+^–H) of an aliphatic Schiff base, formed between chitosan and hydrolyzed acetal group of TGP (Scheme 3, structure 3). The possibility of chitosan cross-linking via hydrolyzed acetal group was earlier demonstrated in [35]. Thus, both types of cross-linkages (Scheme 3, structures 2 and 3) can be realized in the reaction between chitosan and TGP.

The absence of the bands at 911 and 839 cm^−1^ characteristics for the epoxy ring [36] in the FT-IR spectrum of cryogel has confirmed the completeness of the epoxy-ring opening reaction between chitosan and TGP that is important for potential cytotoxicity of the fabricated scaffolds. The completeness of the cross-linking reaction was also confirmed by the low cytotoxicity of chitosan-TGP cryogel (Section 3.4), since unreacted glycidyl ethers demonstrated relatively high cytotoxicity [20].

### 3.3. Morphology and Mechanical Properties of Chitosan Cryogels Cross-Linked with TGP

To understand how the branching of glycidyl ether affects the mechanical properties of chitosan cryogels, we have investigated the mechanical properties of chitosan-TGP cryogels cross-linked at TGP:chitosan mole ratios of 1:4 and 1:12 in comparison with earlier reported cryogels cross-linked with diglycidyl ethers of 1,4-butandiol (BDDGE) [20] and poly(ethylene glycol) (PEGDGE) [21], which supported cell growth in 3D culture.

**Figure 2:**
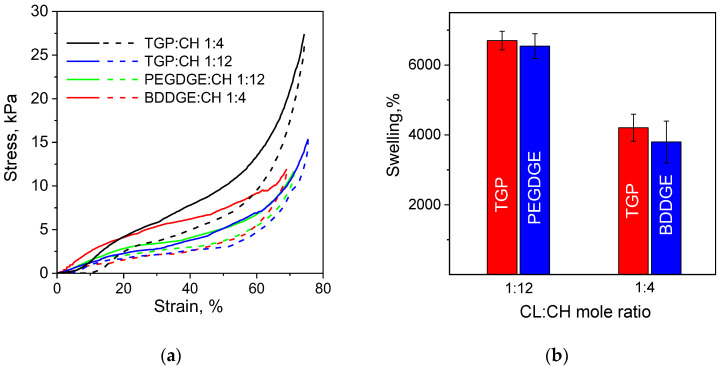
Strain–stress curves of the swollen chitosan (CH) cryogels cross-linked with TGP, BDDGE, and PEGDGE at cross-linker (CL):CH molar ratios of 1:4 and 1:12 (**a**). Equilibrium swelling of the cryogels in water (**b**).

Compressive stress and elasticity of cryogels at 70% strain were calculated from the stress–strain curves recorded under uniaxial compression of the completely swollen cylindrically shaped samples. Strain–stress curves for chitosan cryogels have typical for the biological tissues non-linear shape with strain-stiffening behavior (Figure 2) and hysteresis loop area dependent on the type of cross-linker. The hysteresis observed for all cryogels can be related to the partially irreversible breakage of the primary network of semi-rigid chitosan in the first loading cycle. The ratio of the loop area between the loading and unloading curves to the area under the loading curve (percentage of dissipated energy) was calculated and used as a measure of the cryogels elasticity.

Table 1 shows that elasticity increased in the following order of the cross-linkers PEGDGE > TGP > BDDGE. At low cross-linking density TGP, as a short-armed branched cross-linker, did not demonstrate a significant advantage in terms of elasticity and compressive strength of the obtained scaffolds over the long-chained PEGDGE with M~500. However, PEGDGE application for the fabrication of scaffolds with higher cross-linking density and, thus, higher compressive strength is limited by the high swelling of the hydrophilic PEG blocks [19], which reduces the free volume available for the cell growth inside pores of the swollen cryogel and makes it less suitable for developing 3D cell models.

Permeable chitosan cryogels with higher compressive strength can be obtained using BDDGE but this cross-linker yields the most brittle scaffolds among studied cryogels (Table 1) with ~50% energy dissipated in the first compression cycle due to the emerged structural defects. At high cross-linking density, branched structure of TGP, which was beneficial for the formation of stronger hydrogels at RT (Figure 1a), showed a significant advantage for the fabrication of flexible cryogels. TGP-cross-linked scaffold with high compressive strength of 21 kPa had relatively low percentage of energy dissipated in the compression cycle (Table 1).

All above-discussed cryogels had macroporous structures. The porosity of chitosan-BDDGE and chitosan-PEGDGE was investigated in detail earlier and reported in [20,21]. Porosity of chitosan-TGP cryogels in comparison with chitosan-PEGDGE and chitosan-BDDGE cryogels is demonstrated in Section 3.4. It should be also mentioned that incomplete recovery of the chitosan cryogels height in one cycle (Figure 2) can be related to the loss of the free-flowing water, which was released upon loading and soaked up upon unloading stress due to the action of the capillary forces [37]. Thus, we can conclude that in comparison with earlier reported chitosan cryogels, TGP as a cross-linker provides an additional tool to increase the compressive strength of the scaffold without sacrificing its flexibility. Since the difference in mechanical properties of chitosan-PEGDGE and chitosan-TGP cryogels cross-linked at a molar ratio of 1:12 was not significant, we have selected chitosan-TGP (1:4) to investigate the effect of the scaffold characteristics on growth, morphology, and viability of tumor cells.

### 3.4. 3D Cell Culturing

Recently, we have shown that micro-tumor morphology formed by colorectal cancer cells cryogels can be controlled by the selection of the polymer for the scaffold fabrication: negatively charged carboxymethyl chitosan (CMC) provided microenvironment with low cell-matrix interactions stimulating the formation of cell spheroids, while chitosan supported cell–matrix interaction and guided cell growth as epithelia-like sheets [20]. Considering the large difference in stiffness and elasticity of chitosan-TGP cryogels in comparison with scaffolds cross-linked with PEGDGE and BDDGE, we have investigated how the type of cross-linker affect cytotoxicity of cryogels and morphology of the 3D cell aggregates formed in different scaffolds under the same culturing conditions.

**Figure 3:**
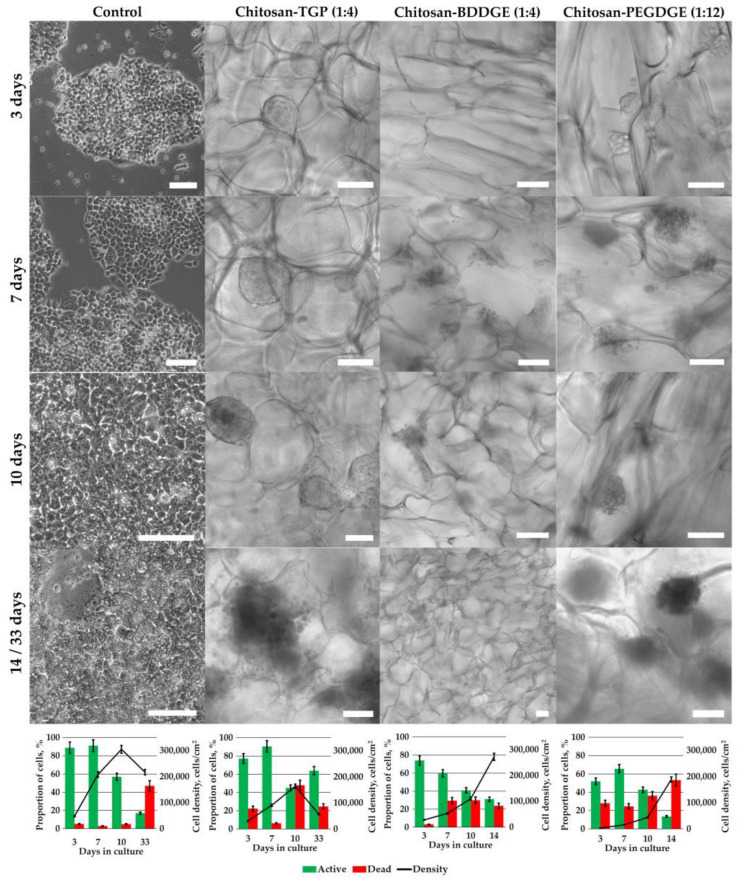
The results of phase-contrast microscopic observation and flow cytometrical analysis of HCT 116 cells cultivated for 3, 7, 10, and 14 days in chitosan-BDDGE (1:4) and chitosan-PEGDGE (1:12) and for 3, 7, 10, and 33 days in chitosan-TGP (1:4) cryogels. The cells were stained with H_2_DCFDA to assess the mitochondrial activity, and DAPI to stain dead cells. The data are presented as the mean of three independent experiments. Standard deviations did not exceed 5%. Scale bar—100 µm.

Human colorectal carcinoma cell line HCT 116 cultured in adhesive plates spread on polystyrene during the first 24 h and started to proliferate reaching cell density of 15,000 cells/cm^2^ by 3rd day, about 200,000 cells/cm^2^ by 7th day, and about 300,000 cells/cm^2^ by 10th day (Figure 3). Then the overgrowth started with an abrupt decrease in the proportion of active cells and density that is normal process in adhesive cultures induced by cell–cell contact inhibition and deficiency of nutrients [38].

In chitosan-TGP (1:4), cryogel the cell growth was slower than in adhesive plate, which is common for 3D cultures and some cell lines have been shown to proliferate 7-fold slower than in 2D culture [39]. The cells grew as dense spheroids of about 100 µm in diameter tightly attached to the cryogel walls and visible by the third day of cultivation. The proportion of active cells was higher than 90%. After the seventh day, the viability started to decrease, but the cell density continued to grow to its maximum of about 170,000 cells/cm^2^ by day 10, probably caused by a complicated removal of dead cells from the pores when replacing the medium. At this stage, the spheroids have their maximal sizes of about 200 µm with the cells experiencing an excess of metabolites accumulated in cryogel pore by neighbor cells and a deficiency of nutrients, which led to a decrease in cell density after the 10th day.

Although the dynamic of HCT 116 cell growth in elastic chitosan-TGP (1:4) cryogel was similar to that in brittle chitosan-BDDGE (1:4) cryogel, there was a pronounced difference in morphology of cell aggregates formed in these scaffolds. In chitosan-BDDGE cryogel, the cells did not form spheroids inside pores as in chitosan-TGP cryogel, they preferred to spread out on the inner surface of the pore walls, forming layered structures, which we have earlier investigated using confocal laser scanning microscopy [20]. Continuously increasing the cell density in chitosan-BDDGE (1:4) cryogel for all 14 days of cultivation, despite a decrease of active cells proportion from about 70% on the 3rd day to almost 40% on the 10th day and 30% on the 14th day, can be explained by the fact that the cells were tended to tightly adhere to the chitosan-BDDGE cryogel surface even being dead. In contrast, in chitosan-TGP (1:4), cryogel by the 33rd day the spheroids became loose, and cells grew mainly on the cryogel surface demonstrating a higher proportion of viable cells than on the 10th day.

There was more similarity in the morphology of cell aggregates in chitosan-TGP (1:4) and chitosan-PEGDGE (1:12) cryogels, which have comparable elasticity (Table 1). However, a much higher swelling degree and lower compressive strength of chitosan-PEGDGE (1:12) cryogel probably affected both cell–cell and cell–matrix interactions. As a result, spheroids had a looser structure than in chitosan-TGP cryogel (Figure 3).

Thus, we can conclude that flexible chitosan-TGP (1:4) cryogel has low cytotoxicity and supports the growth of HCT 116 multicellular structures with morphology more similar to that in highly elastic cryogels of CMC [20]. However, in the former case, large contact area between spheroids and cryogel walls suggests stronger cell–matrix interactions, most likely, due to the presence of free chitosan amino groups at the surface, which are known to promote cell adhesion [40].

## 4. Conclusions

We have suggested a new method of 1,1,3-triglycidyloxypropane (TGP) synthesis via the successive treatment of acrolein with allyl alcohol and further conversion of the obtained 1,1,3-triallyloxypropane to triglycidyl ether. The efficiency of TGP as a cross-linking agent for chitosan under subzero temperature was compared with that of 1,4-butandiol (BDDGE) and poly(ethylene glycol) (PEGDGE). We have shown that TGP yielded scaffolds with higher compressive strength in comparison with cryogels cross-linked with diglycidyl ethers. Investigations of the cross-linking mechanism revealed the heterofunctional nature of TGP, which formed with chitosan covalent bonds not only via epoxy-ring opening reactions but also via interactions of amino groups with hydrolyzed acetal groups. TGP-cross-linked chitosan cryogels possessed low cytotoxicity in relation to HCT 116 cells and supported the formation of 3D multicellular structures of the spherical shape and size up to 200 µm viable for at least 33 days, while in more brittle chitosan-BDDGE cryogel this cell culture formed epithelia-like sheets with lower cell viability. Thus, the choice of cross-linker and cross-linking density can be potentially used to tune properties of chitosan scaffolds and mimic solid tumor microenvironment in vivo in long-term experiments modeling anticancer drugs efficiency, cancer progression, and cell–cell interactions in 3D cell cultures.

## Figures and Tables

**Table 1:** Mechanical properties of chitosan cryogels.

Cross-Linker (CL)	CL:Chitosan Mole Ratio	Dissipated Energy ^1^, %	Compressive Strength ^1^, kPa
BDDGE	1:4	49.4	12.5
TGP	1:4	29.5	21.0
TGP	1:12	35.0	10.7
PEGDGE	1:12	22.2	10.6

^1^ Calculated for 70% strain compression cycle.

## Data Availability

Data are available upon request.

## Supplementary Materials

The following supporting information can be downloaded at: https://www.mdpi.com/article/10.3390/biomimetics8020228/s1, Figure S1: The algorithm of analysis of flow cytometrical data of stained cells.
