# Supplementary material for: Chitosan Cryogels Cross-Linked with 1,1,3-Triglycidyloxypropane: Mechanical Properties and Cytotoxicity for Cancer Cell 3D Cultures
Source: Biomimetics (Basel). 2023 May 29;8(2):228. doi: 10.3390/biomimetics8020228 (PMC10296740; doi:10.3390/biomimetics8020228)
Supplement: Supplementary file 1 [file biomimetics-08-00228-s001.zip › biomimetics-2400685-supplementary.pdf]

Supplementary materilas

Chitosan cryogels cross-linked with 1,1,3-triglycidyloxypropane: mechanical properties and cytotoxicity for cancer cell 3D cultures

Yuliya Privar, Andrey Boroda, Alexandr Pestov, Daniil Kazantsev, Daniil Malyshev, Anna Skatova and Svetlana Bratskaya

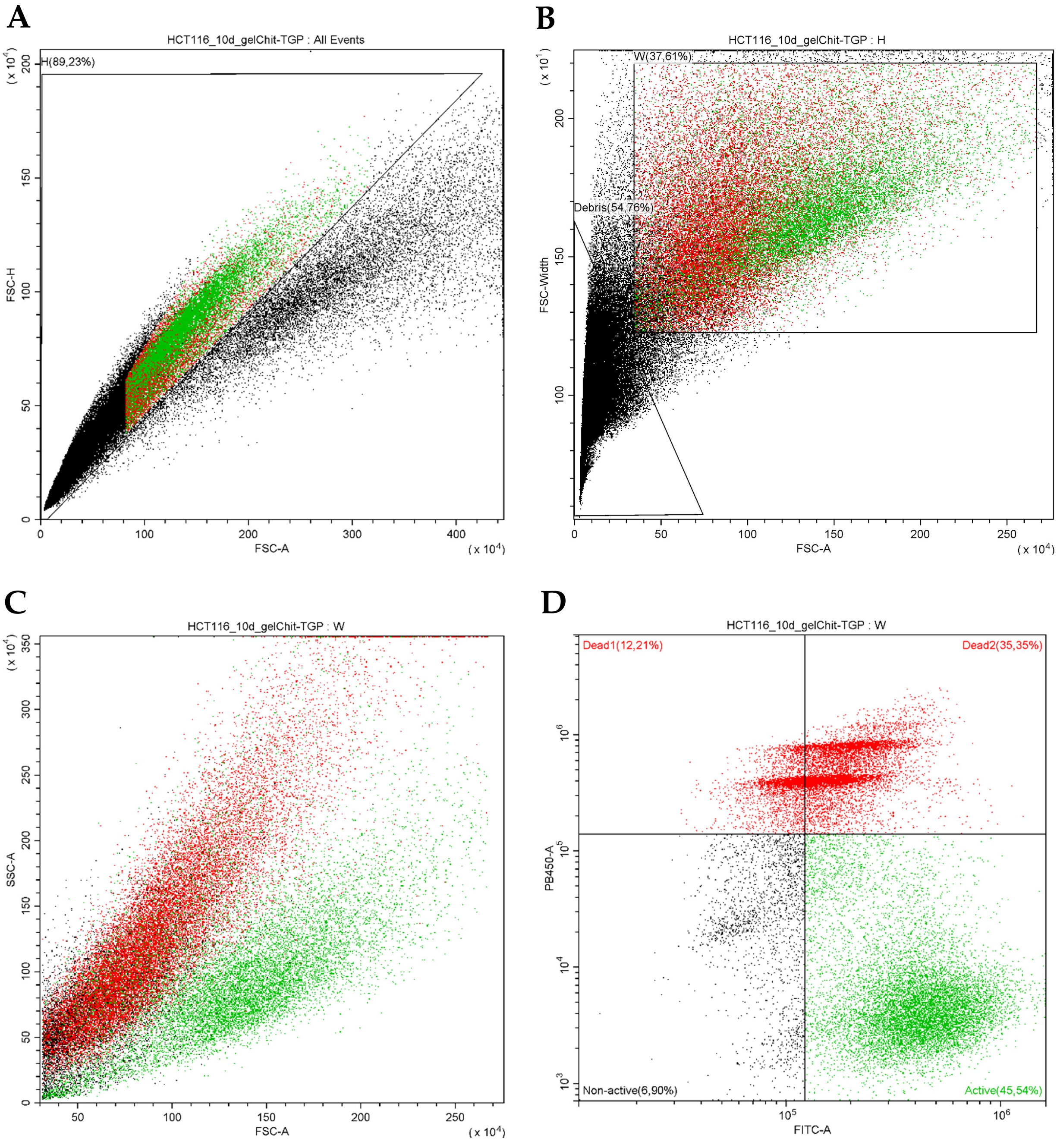

Figure S1. The algorithm of analysis of flow cytometrical data of stained cells. The presented example are cells from spheroids cultivated in cryogel disk of chitosan-TGP (1:4) for 10 days, disaggregated and detached from the disk, and stained for 10 min at room temperature in the dark with 10 μM 2′,7′-dichlorodihydrofluorescein diacetate (excitation in FITC fluorescent channel) to assess the mitochondrial activity, and 1 μg/mL 4′,6′-diamidino-2-phenylindole (PB450 fluorescent channel) to stain dead cells.

Single events (about 90% of total events in each sample) were determined by triangle gating on FSC-A against FSC-H plot in order to exclude cell aggregates from the following analysis (A). The gate of analyzed cells ("W") was separated from debris by gating on FSC-A against FSC-W (B). FSC-A against SSC-A plot represents the relative size and granularity of events, respectively (C). The proportion of dead cells (4′,6′-diamidino-2-phenylindole-positive events) is determined as a sum of "Dead1" and "Dead2" gates from the chosen at the previous step gate "W" (D). The proportion of active cells includes all 2′,7′-dichlorodihydrofluorescein diacetate-positive events from the chosen at the previous step gate "W" (D).
